# Review of clinical approaches in fluorescence lifetime imaging ophthalmoscopy (Erratum)

**DOI:** 10.1117/1.JBO.23.9.099802

**Published:** 2018-09-27

**Authors:** Lydia Sauer, Karl M. Andersen, Chantal Dysli, Martin S. Zinkernagel, Paul S. Bernstein, Martin Hammer

**Affiliations:** a University Hospital Jena, Jena, Thuringia, Germany; b University of Utah, John A. Moran Eye Center, Salt Lake City, Utah, United States; c Geisinger Commonwealth School of Medicine, Scranton, Pennsylvania, United States; d Bern University Hospital, Inselspital, Department of Ophthalmology, Bern, Switzerland; e University of Jena, Center for Biomedical Optics and Photonics, Jena, Germany

## Abstract

This erratum corrects an error in “Review of clinical approaches in fluorescence lifetime imaging ophthalmoscopy”

This article [*J. Biomed. Opt.* **23**(9), 091415(2018)] was originally published online 4 September 2018, with an error involving the images presented for Figs. 6 and 7. The image presented for Fig. 6 should have been presented for Fig. 7, and vice versa. All captions and textual references involving these figures were otherwise correct.

The images for Figs. 6 and 7 in this article were corrected online on 7 September 2018.

They appear correctly in print.

